# Motion control of thumb and index finger of an artificial hand for precision grip using asynchronous decoding of CM cell activity

**DOI:** 10.1186/1471-2202-14-S1-P254

**Published:** 2013-07-08

**Authors:** Selim Eskiizmirliler, Olivier Bertrand, Michele Tagliabue, Marc A Maier

**Affiliations:** 1 CNRS UMR 8194, University Paris Descartes, Sorbonne Paris Cité, Paris, F-75006, France; 2 University Paris Diderot, Sorbonne Paris Cité, Paris, F-75013, France

## 

Recent brain machine interface (BMI) applications in humans [1] have shown the particular benefits of decoding of neural signals and of their use in motion control of artificial arms and hands for paralyzed people. Despite of the spectacular advances in the last decade on the kinematic control of reach and grasp movements, the dynamic control of these movements together with the choice of powerful decoding algorithms continues to be a major problem to be resolved. This work reports the results on asynchronous decoding, obtained with the application of artificial neural networks (ANN) [2], to both thumb and index finger kinematics and to their EMG. Neural data (spike trains and EMG) were recorded in the monkey. This work aims at providing a complete BMI framework to reproduce precision grip like hand movements with our 2 finger artificial hand (see Figure.1.A and B).

The database was composed of up to six simultaneously recorded CM cell activity, of up to nine EMGs from different forearm muscles, and of the two fingertip position recorded in two monkeys while they performed a precision grip task (see Figure.1.A for robot reproduction). The CM cell activities were used as inputs and thus to train the time-delayed multi-layer perceptron (TDMLP) associated to each recording session in order to estimate both EMG and fingertip position signals. Each training epoch was performed with five different sliding window lengths that also determine the length of the input vector (i.e. the number of spikes considered at each instant) belonging to the time interval [25 ms,..,400 ms]. We trained the networks following three different paradigms: 1) Training the ANNs with only one spike train (from one CM cell). 2) Training the networks with all simultaneously recorded spike trains. 3) Only for the EMG estimation: training the networks with identified and non-identified CM cell spike trains. The identity of a cell as a CM cell was defined by the presence of a post spike facilitation (PSF) obtained by spike triggered averaging of the EMG. We then analyzed statistically the effects of each parameter on the estimating performance of the ANNs.

Finally, we used the fingertip position signals estimated by the corresponding trained ANN to drive the index (4 DoF) and thumb finger (5 DoF) of our artificial hand (Shadow Robot Company **©**). We then compared the reproduction performance of the hand to the recorded and estimated position signals (Figure 1B).

**Figure 1:**
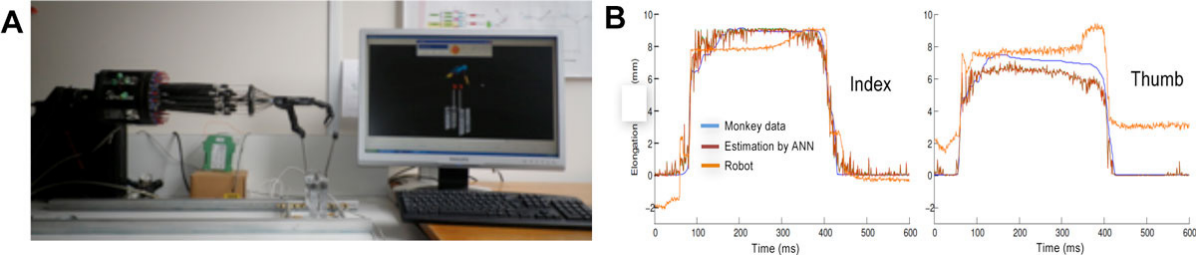
**A. Artificial forearm with a 2-finger hand of the precision grip setup actuated by pneumatic muscles, B. Reproduction of thumb and index finger position**.

## Conclusion

The current results confirmed that the effects of the sliding window length, as well as that of the parameters of each of the three learning paradigms, reported for the index finger only [2], are also valid for the thumb data. This suggests that the developed BMI system using the same TDMLP structures can be used to control the concerted action of the two fingers to reproduce precision grip or precision grip-like hand movements.
